# Removal of Microplastics in a Hybrid Treatment Process of Ceramic Microfiltration and Photocatalyst-Mounted PES Spheres with Air Backwashing

**DOI:** 10.3390/membranes14080169

**Published:** 2024-07-31

**Authors:** Minjoo Song, Jin Yong Park

**Affiliations:** Department of Environmental Sciences & Biotechnology, Hallym University, Chuncheon 24252, Republic of Korea; smj980324@gmail.com

**Keywords:** microplastics, titanium dioxide, polyether sulfone, air backwashing, ceramic, microfiltration, water treatment

## Abstract

Microplastics (MPs), which are defined as plastics with a size of less than 5 mm, cannot be treated completely in wastewater treatment plants (WWTPs) and discharged to a water body because they are too small in size. It has been reported that MPs can have adverse effects on human beings and water ecosystems. There is a need to combine existing drinking water treatment plants (DWTPs) and WWTPs with the traditional treatment process and technology with high removal efficiency of MPs or to develop a new technology to separate MPs from water and wastewater. In this study, the effects of MPs (polyethylene (PE), 125 μm) and organic matter (humic acid) were researched in a hybrid treatment process of ceramic microfiltration (MF) and photocatalyst (TiO_2_)-mounted polyether sulfone (PES) spheres with air backwashing. The roles of the MF, photooxidation, and adsorption of PES spheres were confirmed in a single MF process (MF), an MF process with UV irradiation (MF+UV), MF and PES sphere adsorption without UV irradiation (MF+PES), and a hybrid process incorporating MF and PES spheres with UV irradiation (MF+PES+UV). The impact of the air backwashing cycle (filtration time, FT) on filtration characteristics and treatment efficiencies in the hybrid process was studied. In the MF process, membrane fouling increased with increasing organic matter (HA, humic acid). The treatment efficiency of MPs increased; however, that of dissolved organic matter (DOM) decreased with increasing HA. As MPs increased, the membrane fouling decreased; however, total filtration volume (V_T_) remained almost constant. The treatment efficiency of MPs increased a little, and that of DOM showed a dropping trend. In the hybrid process, the membrane fouling was controlled via the adsorption and UV photooxidation of the PES spheres, and the DOM treatment efficiency increased by combining processes from MF to MF+PES+UV. The optimal FT was 10 min at BT 10 s in this hybrid process. The results could be applied to separate MPs effectively in DWTPs/WWTPs.

## 1. Introduction

Since the invention of plastics, the amount of plastic used by humans has increased every year and the number of MPs generated has increased rapidly. MPs are new pollutants caused by the decomposition of various types of plastics during the use and disposal of plastics [1]. MPs are small plastics with a size of less than 5 mm and can be divided into primary and secondary plastics according to the cause of their occurrence. Primary plastics are intentionally manufactured for specific applications, such as toothpaste, spheres used in face washes and cosmetics, and industrial abrasives. Secondary plastics are produced via the decomposition of large plastics due to environmental factors such as ultraviolet radiation, physical wear, and oxidation [2]. MPs found in river water have been observed in various shapes such as pellets, fibers, and debris [3]. Today, MPs are found in water systems around the world, including marine and freshwater environments, which are sources of drinking water for human consumption [4]. Looking at the current status of MPs found in Korean freshwater, the MP concentration of the surface water of the Han River was 20~180 particles/m^2^, and that of its tributaries was 1.2~234.5 particles/m^2^ [5]. The mean (±standard deviation) MP content of Nakdong River was 293 ± 83 (upstream, February 2017)~4760 ± 5242 (downstream, August 2017) particles/m^3^ in water and 1970 ± 62 particles/kg in sediment. Polyethylene (PE), Polypropylene (PP), and Polystyrene (PS) were the most common MPs identified. MPs smaller than 300 μm in size accounted for 74% of the MPs in water and 81% in sediment, with the largest distribution ranging in size from 50 to 150 μm [6]. The dangers of MPs to the human body have not yet been clearly understood, but in the last 5 years, studies have been published one after another on the possibility of adverse effects on the human body if MPs enter the body, and MPs are one of the most actively researched topics. A survey that shows the number of published research papers on MP pollution in each water system shows that research on MP pollution in water systems has continued to increase over the past decade [7]. MPs with a size of 100 nm or less can reach almost all organs after entering the human body, so there is concern about the adverse effects of MPs continuously accumulating in the human body [8]. A report published by UNEP in 2021 suggested the impact of microplastics on human health by body part [9]. According to that report, MPs cause various cardiovascular, respiratory, and thyroid diseases and various problems in pregnant women and fetuses. In addition, if MPs accumulate in the body of marine organisms, it can cause malnutrition, internal organ damage, inflammation, decreased fertility, and death, and ecotoxicity can be caused by the plastic itself, reaction by-products, and additives [10].

Most MPs can be removed through conventional DWTPs and WWTPs, but some are still untreated and discharged back into the water system. Looking at the incoming route of MPs and the steps of water treatment in WWTPs, there is no distinct process for MP removal in WWTPs yet. Therefore, there is a need to combine the existing DWTPs and WWTPs with the traditional treatment process and technology, with a high removal efficiency of MPs, or to develop a new technology to separate MPs from water and wastewater. According to a literature analysis, the membrane process, which is one of the most advanced treatment technologies, may be the most effective and promising tool for removing MPs [11]. Among the membranes, ceramic membranes have the advantages of being chemically and physically strong and having a long lifetime, a higher resistance to backwashing pressure and cleaning agents than polymer membranes, and a small fouling rate. In addition, the membrane process is more efficient than other advanced processing techniques because of a smaller pore size than MPs [12].

In a domestic and international study that attempted to remove MPs using membranes, H. Takeuchi et al. [13] combined activated sludge and ceramic membrane processes on a laboratory scale and removed more than 96% of the MPs. B. Ma et al. [14] analyzed the removal behavior of PE in flocculation and ultrafiltration (UF) processes. J. Yang et al. [15] determined the MP removal efficiency by analyzing water samples from WWTPs, DWTPs, and seawater treatment plants (SWTPs) and removed about 92% of the MPs using UF membranes. A. Mohana et al. [16] effectively removed nanoparticles (NPs) and MPs using metal–organic framework (MOF)-based UF membranes. L. Li et al. [17] confirmed the performance of removing low-density micro-particulates such as MPs through the formation of a dynamic membrane on the surface of the support membrane. S. Ziajahromi et al. [18] characterized and quantified MPs in WWTP effluent using the reverse osmosis (RO) process. J. Talvitie et al. [19] removed about 99% of MPs in the wastewater treatment process using a membrane bioreactor (MBR).

A lot of research has been conducted to develop TiO_2_ photocatalysts capable of degrading MPs. N-doped TiO_2_ powder showed a 6.4% treatment rate of HDPE [20]. Mesoporous N-TiO_2_ prepared via evaporation-induced self-assembly showed a 56% mass loss [21]. TiO_2_ nanoparticle film achieved PS and PE degradation, and the weight loss was over 98% [22]. C,N-doped TiO_2_ powder showed remarkable performance for HDPE, and the weight loss was 71.77% [23]. Degussa P25 (TiO_2_) was employed for the photocatalytic degradation of polyamide 66 (mass loss of 97%) in wastewater treatment plants [24]. TiO_2_ prepared via the electrochemical anodization technique for Ti foils displayed a 23.50% elimination of PS [25]. Ag/TiO_2_/RGO prepared via photoassisted deposition and ultrasonic radiation showed a 76% degradation of PE [26]. Nano-Composite Ag/TiO_2_ synthesized via photoassisted deposition offered a 100% PE degradation [27]. TiO_2_ in amylose, polyiodide, and hydroxyethyl cellulose achieved a weight loss of over 15% for LDPE [28]. C,N-TiO_2_/SiO_2_ semiconductors achieved the degradation of the PET MPs (mass losses of 9.35 and 16.22%) [29]. With the comprehensive action of the nano-flower-shaped N-doped TiO_2_ catalyst (Pt@N-TiO_2_-1.5%) on the relatively low-molecular-weight intermediates, an approximate 29% weight loss was induced on the pretreated PET-FMPs [30]. Self-asymmetric Pac-Man TiO_2_ degraded up to 28% of PS particles after 70 h of UV light exposure [31]. A hybrid TiO_2_ synthesized using a waste natural resin as a component without any chemical modification induced the oxidative degradation of LLDPE under indirect daylight [32].

In this study, the impact of organic matter and MP concentrations on membrane contamination was confirmed in the tubular ceramic MP process alone. In addition, in the hybrid water treatment process using tubular ceramic MFs and photocatalyst-mounted PES spheres, the role of adsorption and photooxidation for each process and the membrane filtration characteristics and treatment efficiency according to the air backwashing cycle were identified, and optimal operating conditions were derived.

## 2. Materials and Methods

The ceramic microfiltration membrane used in this study was the tubular MF membrane NCMT-7231 purchased from Nano Pore Materials Co., Ltd. (Seoul, Korea), which is coated with α-alumina material on the same α-alumina support layer. The specifications of the ceramic membrane used in this research are presented in Table 1.

**Scheme 1:**
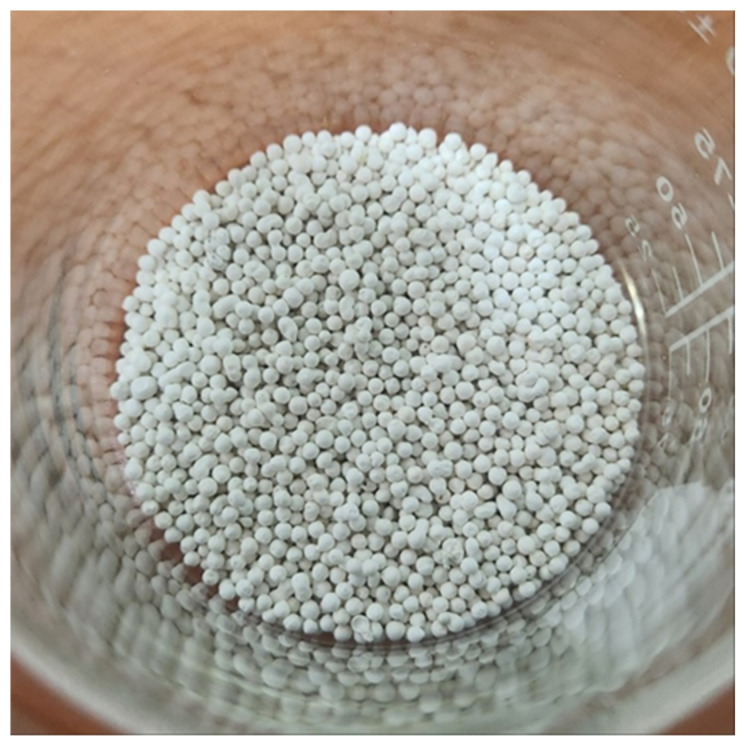
PES microspheres mounted with TiO_2_ photocatalyst.

The photocatalysts used in this study were PES microspheres manufactured by E. Drioli [33] via the phase inversion of a mixture of titanium dioxide (TiO_2_) powder and polyether sulfone (PES), and the diameter of each sphere was 1.2–1.4 mm. The weight percent of TiO_2_ was 30% in the PES microspheres. Scheme 1 shows the TiO_2_-mounted PES spheres used in this research.

In order to simulate the humic substances, which account for a considerable amount of the natural organic matter subject to water purification, and MPs, the model solution was prepared using humic acid and polyethylene (PE) powder. The model solution was prepared at a certain concentration by mixing an amount of HA (humic acid sodium salt, Sigma-Aldrich, St. Louis, MO, USA) and PE (Sigma-Aldrich, St. Louis, MO, USA) powder with an average particle size of 125 μm in distilled water. The reason for using the 125 μm sized MPs is that the size of the MPs found in fresh water in Korea is mostly distributed in the 50–150 μm range, and one of the most common MPs found is PE.

**Figure 1:**
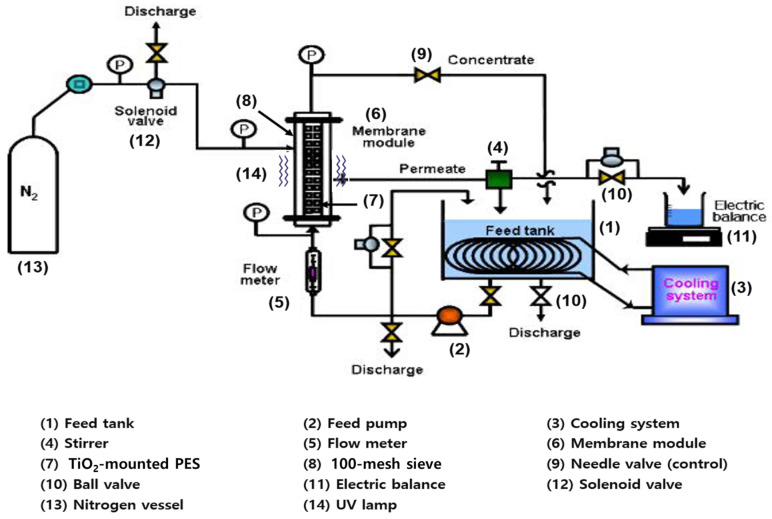
Apparatus of advanced water treatment system using hybrid module of tubular ceramic MF (NCMT-7231) and TiO_2_-mounted PES spheres with periodic air backwashing.

Figure 1 is a microfiltration diagram showing the cross-flow filtration method, which can backwash treated water, used in this study. For the removal of MPs and dissolved organic matter (DOM), the PES spheres (7) mounted with TiO_2_ powder via the phase inversion method were filled between the outside of the membrane and the inside of the module, and it was composed in the form of a single module (6). In addition, the treated water, which passed through the ceramic membrane and the PES sphere mounted with TiO_2_, flowed through the upper outlet of the installed module with a mesh size of 8 of 100 (0.150 mm), which is much smaller than the PES sphere, to prevent the PES spheres in the membrane module from being washed out along the treated water.

**Scheme 2:**
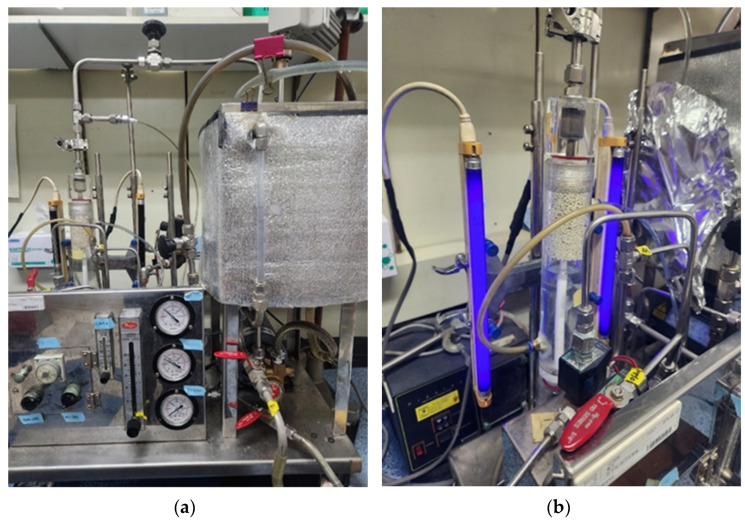
Advanced water treatment system using a hybrid module of tubular ceramic MF (NCMT-7231) and TiO_2_-mounted PES spheres with periodic air backwashing; (**a**) control board (**b**) membrane module and UV lamps in the hybrid water treatment system.

Two UV lamps (14) (F8T5BLB, Sankyo, Kanagawa, Japan) were installed on the outside of the module and irradiated UV of 352 nm, which was the most effective wavelength to decompose DOM, during the experiment. The feed tank (1) was a stainless steel tank with a capacity of 20 L, and it was installed by connecting the copper pipe coil with the cooling water (3) to maintain a constant temperature of the feed model solution. In addition, a stirrer (4) was installed in the center of the upper part of the feed tank to continuously stir the feed solution so that it could be supplied in a homogeneous state. The feed solution was introduced into the membrane module from the feed tank using a pump (2) (Procon, Standex Co., Salem, NH, USA), and the inflow flow rate was measured using a flow meter (5) (NP-127, Tokyo Keiso, Tokyo, Japan). At the inlet and outlet parts of the membrane module, pressure gauges measuring up to 6.8 bar were installed to check the pressure difference in the membrane. The flow rate and pressure of the membrane module were kept constant by operating the valves (9) at the bypass of the pump (2) and the concentrated water lines, and the mass of the permeate water was treated using the ceramic membrane and the PES spheres were measured with an electronic scale (11) (Ohaus, Canton, MA, USA) to obtain the permeate flux. The treated water was circulated to the feed tank (1) to keep a persistent feed concentration during experimentation. In order to control FT and BT, the permeate water outlet, pump bypass pipe, and nitrogen inflow line were equipped with solenoid valves (12) (CKD, Aichi, Japan). All three solenoid valves were connected to a time controller (twin timer: Omron, Japan) so that the valve opened and closed at the same time, and all the water of the module was discharged through the upper and lower parts of the membrane module via the pressure of the nitrogen tank for backwashing. Scheme 2 are actual device photographs of the hybrid water treatment unit, membrane module, and UV lamps used in the experiment.

To determine the correlation between MP concentration and turbidity, distilled water was mixed with 125 μm particle-sized PE powder (MPs), and the turbidity was measured by varying PE concentrations to 0.5, 1, 2, 5, 10, and 15 mg/L. In the tubular ceramic microfiltration (NCMT-7231) process alone, the effect of organic matter on membrane fouling was checked by changing the concentration of humic acid to 10, 15, 20, and 25 mg/L at a constant concentration of 1 mg/L of MPs in the model solution. The pH of the model solution was 6.7~6.8. Using a model solution with an MP concentration of 2 mg/L and HA of 20 mg/L, the roles of the microfiltration, adsorption, and photooxidation of the photocatalyst-mounted PES spheres were examined through the ceramic microfiltration process (MF), the MF and the PES sphere process without UV irradiation (MF+PES), the MF and UV irradiation process without a photocatalyst (MF+UV), and the MF and photocatalytic-mounted PES sphere process with UV irradiation (MF+PES+UV). Lastly, in the hybrid water treatment process using ceramic microfiltration and photocatalyst-mounted PES spheres with UV irradiation, the optimal operation conditions for BT at NBF (no backwashing) were 6, 10, 15, 20, and 30 s, and this was examined at a constant 10 min FT with the model solution of the MP concentration of 2 mg/L and an HA concentration of 20 mg/L. In addition, the effect of backwash cycle FT was observed by keeping the BT constant at 10 s and changing the FT to NBF, at 6, 8, 10, 20, and 30 min. In all experiments, the transmembrane pressure (TMP) was maintained at 1.80 bar, the backwash pressure was 2.50 bar, the inlet flow rate was 1.0 L/min, and the feed water temperature was maintained at 20 ± 1.0 °C. The operation was performed for 180 min under each condition, and changes in the resistance of membrane fouling (R_f_), permeate flux (J), initial permeate flux (J_0_), dimensionless permeate flux (J/J_0_), and total filtration volume (V_T_) were detected. After finishing each experiment after 180 min, a physical cleaning using a small brush was performed inside the membrane tube. For estimating the resistances of irreversible and reversible membrane fouling (R_if_ and R_rf_), the treated water flux was checked after the physical cleaning.

At the end of the experiment, the model solution used as feed water was drained, and the distilled water was circulated for about 1 h to clean the experimental device and membrane. Then, after separating and collecting the TiO_2_-mounted PES spheres from the membrane module, the ceramic membrane was removed from the module. The membrane was desorbed and chemically cleaned in 0.25 N aqueous sodium hydroxide solution and 15% aqueous nitrate solution for 1 h and 1 day, respectively. After that, it was heated in a furnace at 550 °C for 1 h, cooled naturally for 24 h, and stored in distilled water. Before starting the experiment under other conditions, the membrane was installed in the module and the permeate flux was measured while operating normally with primary distilled water to check whether the performance of the membrane was restored. If the permeate flux was restored to less than ±5% of the standard one, the experiment was started.

In order to analyze the water quality of the supply water and treated water to check the treatment efficiency of MPs and DOM, samples were collected from the feed tank and the treated water line every 30 min after the start of operation. Turbidity to determine MP concentration was measured directly using a turbidimeter (2100N, Hach, Loveland, CO, USA). UV_254_ absorbance, an indicator of the DOM-like humic acid, was measured using a UV spectrophotometer (Genesys 10 UV, Thermo, Waltham, MA, USA). A photograph of the inside and outside of the membrane was taken using a Field Emission Scanning Electron Microscope (FE-SEM, JSM-7900F, JEOL, Seoul, Korea) at Kangwon University (Chuncheon, Korea) after operating the microfiltration water treatment processes several times.

## 3. Results and Discussions

### 3.1. Removal of Microplastics in Ceramic Microfiltration Water Treatment Process

#### 3.1.1. Impact of Organic Matter Concentration (Humic Acid, HA)

**Figure 2:**
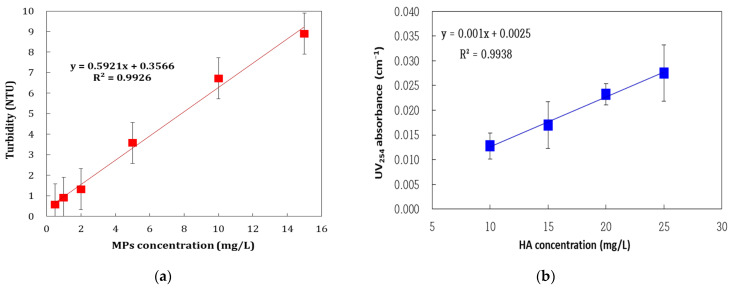
Calibration curve: (**a**) turbidity vs. MPs; (**b**) UV_254_ absorption vs. HA concentration.

To determine the correlation between MP (PE) concentration and turbidity, the turbidity was measured by changing the MP concentrations to 0.5, 1, 2, 5, 10, and 15 mg/L. Figure 2a shows a calibration curve of turbidity for MP concentration. As a result, it was confirmed that the turbidity was proportional as the concentration of MPs increased, and based on these results, the treatment efficiency of MPs was calculated through turbidity measurement. It is difficult to measure turbidity below 0.5 mg/L PE. So, our experimental range of PE concentration was higher than in other research. In addition, to determine the correlation between humic acid (HA) concentration and UV_254_ absorbance, the UV_254_ absorbance was measured by changing the HA concentration to 10, 15, 20, and 25 mg/L. Figure 2b shows the calibration curve of UV_254_ absorbance for HA concentration. As a result, the UV_254_ absorbance increased proportionally as the concentration of HA increased. Therefore, the treatment efficiency of dissolved organic matter (DOM), which was HA in this research, was obtained through UV_254_ absorbance analysis.

The MP concentration of the model solution was kept the same at 1 mg/L, FT for 10 min, and BT for 10 s, during periodic nitrogen backwashing, and the HA concentration was changed to 10, 15, 20, and 25 mg/L and the effect of organic matter was examined. Backwashing with air is a common backwashing method, but the reason why we backwashed with nitrogen instead of air in this study was to minimize the possibility that the oxygen contained in the air would affect water quality. The average values of resistances of the membrane itself (R_m_), the boundary layer (R_b_); the final resistances of membrane fouling (R_f,180_), irreversible fouling (R_if_), and reversible fouling (R_rf_) after 180 minutes’ operation; initial permeate flux (J_0_); permeate flux (J_180_); dimensionless permeate flux (J_180_/J_0_); and the total filtration volume (V_T_) after 180 minutes’ operation are summarized in Table 2, after repeated experiments.

**Figure 3:**
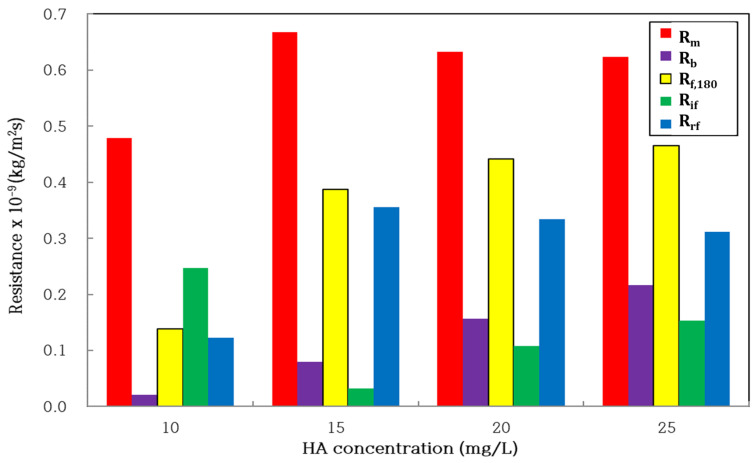
Impact of humic acid concentration on filtration resistances for the process of tubular ceramic MF (NCMT-7231) with air backwashing in a bar graph.

Filtration resistance (R_m_, R_b_, R_f_, R_if_, and R_rf_) and transmission line flux (J) were calculated using resistance-in-series filtration theory (J = ΔP/(R_m_ + R_b_ + R_f_)), with the equivalent means utilized at the former result [34], where ΔP is TMP. After the experiments under each condition, the ceramic membrane was removed from the module, and the inside of the membrane was physically cleaned using a cleaning brush. The membrane was then reassembled into the module to measure the permeate flux of pure water. The R_if_ was calculated by substituting the measured permeate flux into the resistance-in-series filtration equation, and the R_if_ value was subtracted from the R_f,180_ value to determine the R_rf_. Figure 3 compares the values of R_m_, R_b_, R_f,180_, R_if_, and R_rf_ in a bar graph.

In Table 2 and Figure 3, R_m_ and R_b_ are the minimum values, and J_180_ and V_T_ are the maximum at HA 10 mg/L, and the membrane fouling (R_f,180_) is the lowest in this experimental range of HA 10~25 mg/L. R_f,180_ was the highest at the maximum concentration of HA of 25 mg/L under these experimental conditions. However, R_if_ showed a maximum value at HA 10 mg/L and a minimum value at HA 15 mg/L, while R_rf_ showed a minimum value at HA 10 mg/L and a maximum value at HA 15 mg/L.

These results show that membrane fouling worsens as the concentration of organic matter increases. Under HA 10 mg/L condition, irreversible membrane fouling is judged to be the most common due to the infiltration of contaminants into the membrane. On the other hand, it was found that when the concentration was more than 15 mg/L, a thick layer of cake was formed on the surface of the membrane, which increased the reversible membrane fouling, which was easily removed with a brush.

**Figure 4:**
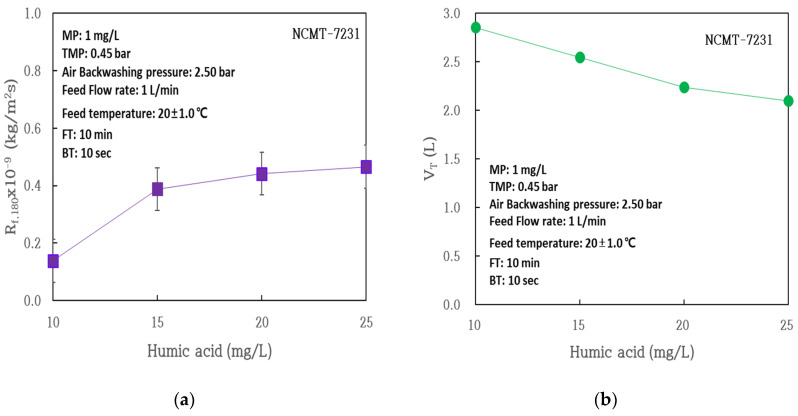
Impact of the humic acid concentration: (**a**) the final resistance of membrane fouling; (**b**) total filtration volume.

In Figure 4, R_f_ and V_T_ were examined according to organic matter concentrations. As shown in Figure 4, R_f,180_ showed a slowly increasing trend after a sharp increase in HA concentrations between 10 and 15 mg/L, while V_T_ showed a continuously decreasing trend. These results are due to the fact that as the concentration of HA increases, the membrane fouling inside and on the surface of the membrane increases.

**Scheme 3:**
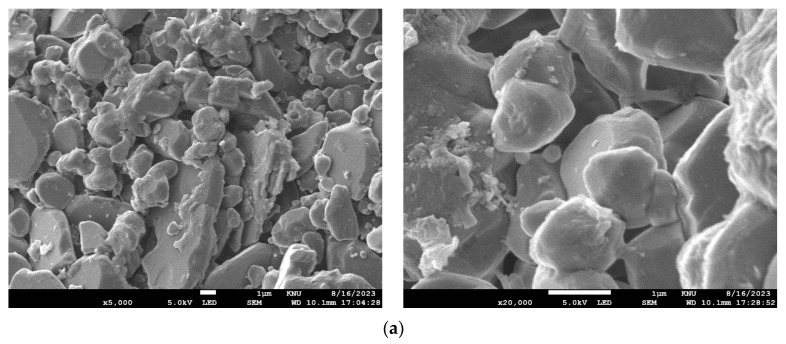

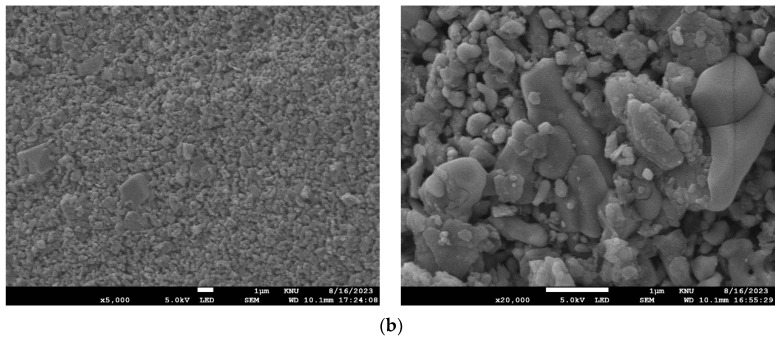
SEM (Scanning Electron Microscope) pictures of the alumina membrane surface: (**a**) Outside surface membrane (×5000 and ×20,000); (**b**) Inside surface membrane (×5000 and ×20,000).

Scheme 3 is a photograph taken with a Field Emission Scanning Electron Microscope (FE-SEM) on the inside and outside of the membrane after operating the microfiltration water treatment processes several times. Looking at Scheme 3, the outer surface of the membrane is almost clean and membrane pores can be distinguished. However, since the membrane module was operated in an in-and-out manner, it was observed that contaminants accumulated on the inner surface of the membrane and the pores were blocked by contaminated particles. These results are consistent with the previous findings of this research team [35].

**Figure 5:**
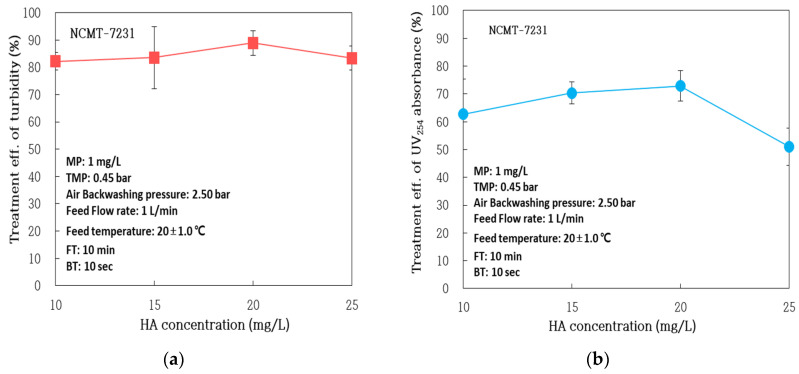
Impact of the humic acid concentration on treatment efficiency: (**a**) turbidity (MPs); (**b**) UV_254_ absorbance (DOM).

Figure 5 shows the treatment efficiency of MPs and DOM according to the concentration of HA.

Even when the concentration of humic acid increased, there was no significant difference in MPs treatment efficiency; however, DOM treatment efficiency tended to decrease. These results imply that high concentrations of dissolved organic matter limit the removal of DOM using the ceramic MF process alone.

#### 3.1.2. Impact of Microplastic (MP) Concentrations

**Figure 6:**
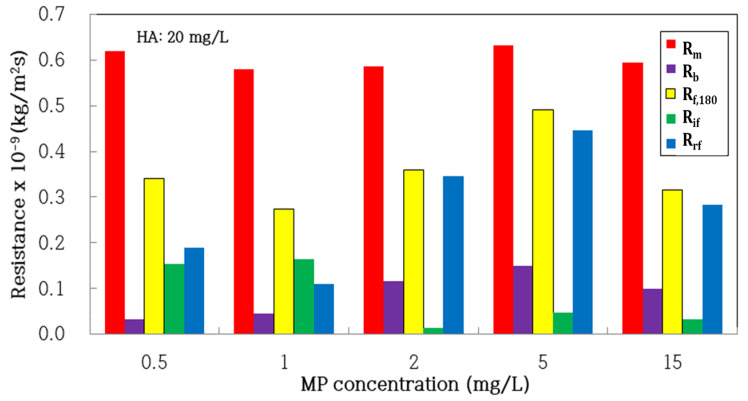
Impact of microplastic concentration on filtration resistances for the process of tubular ceramic MF with air backwashing.

To investigate the effect of MPs on membrane fouling in the tubular ceramic MF process alone, the effects of MPs were examined by changing the concentration of MPs to 0.5, 1, 2, 5, 10, and 15 mg/L at a constant HA 20 mg/L concentration of the model solution. In order to suppress the membrane fouling, periodic air backwashing was performed for 10 s every 10 min, which was identified as the optimal condition. After repeated experiments, the mean values of the filtration factors according to MP concentration were summarized in Table 3. Figure 6 compares the values of all filtration resistances in a bar graph. In Table 3 and Figure 6, the initial permeate flux of pure water was kept constant within the margin of error of 5% before the experiment, so the R_m_ was similar regardless of the MP concentration. R_f,180_ and R_rf_ were the minimum values at MPs concentrations of 1 mg/L and the maximum at MPs 5 mg/L, while J_0_, J_180_, and V_T_ were the maximum at MPs of 1 mg/L.

**Figure 7:**
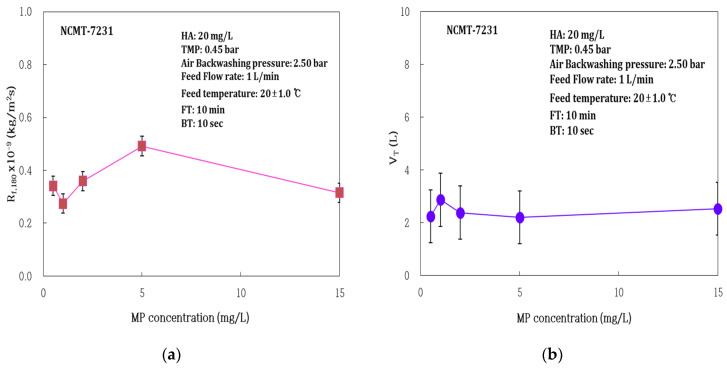
Impact of microplastic concentration: (**a**) final resistance of membrane fouling; (**b**) total filtration volume.

Figure 7 examines the impact of MP concentration on final membrane fouling (R_f,180_) and total filtration volume (V_T_) after 3 h of operation. As shown in Figure 7, the R_f,180_ tended to increase as MP concentrations increased, peaking at 5 mg/L and then decreasing; it showed a minimum value at 1 mg/L MPs. The V_T_ showed the highest value at 1 mg/L of MPs, where there was a minimum of R_f,180_, and it was the lowest at 5 mg/L of MPs, where there was a maximum of R_f,180_. As a result of these findings, the membrane fouling did not intensify significantly even with an increase in MP concentration, and it decreased under the conditions of the highest MP concentration in the scope of this study. The reason for this is that MP particles are considerably larger than the size of the pores, so they do not penetrate into the membrane and cause irreversible membrane fouling; rather, they have the effect of cleaning the membrane surface and reducing membrane fouling.

**Figure 8:**
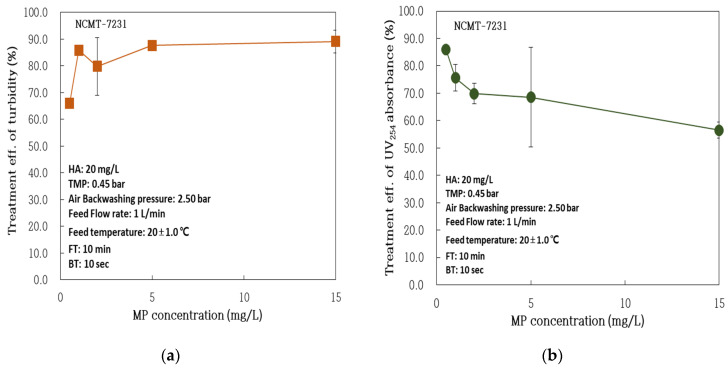
Impact of the microplastic concentration on treatment efficiency: (**a**) turbidity (MPs); (**b**) UV_254_ absorbance (DOM).

Figure 8 shows the treatment efficiency of MPs and DOM according to the concentration of MPs in the ceramic MF process alone. As the concentration of MPs increased, the average treatment efficiency of the MPs (turbidity) tended to increase slightly; however, the treatment efficiency of the DOM (UV_254_ absorbance) showed a clear decreasing trend. As a result of these results, MP treatment efficiency was not significantly related to MP concentration. The reason for the decrease in the treatment efficiency of the DOM seems to be that as the MP concentration increases, the cleaning effect of the membrane surface occurs, which inhibits the formation of the cake layer.

### 3.2. Removal of Microplastics in Hybrid Water Treatment Processes of Ceramic Microfiltration (MF) and Photocatalyst (TiO_2_)-Mounted PES Spheres

#### 3.2.1. Roles of Microfiltration and Photocatalytic Adsorption, Photooxidation in Periodic Air Backwashing

**Figure 9:**
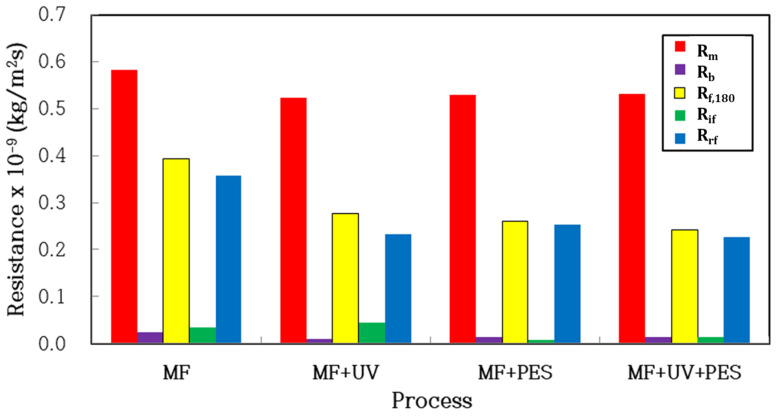
Roles of MF, adsorption, and photooxidation on filtration resistances in the hybrid process of tubular ceramic MF and TiO_2_-mounted PES spheres at PE (MPs) 2 mg/L and HA 20 mg/L.

The roles of the MF, adsorption, and photooxidation of photocatalyst (TiO_2_)-mounted PES spheres were investigated in the process with ceramic MF alone (MF), the process of PES spheres without UV irradiation (MF+PES), the MF process with UV irradiation (MF+UV), and the photooxidation process using ceramic MF and TiO_2_-mounted PES spheres with UV irradiation (MF+PES+UV) in the model solution with PE (MPs) 2 mg/L and HA 20 mg/L concentrations. After repeated experiments, the mean values of the filtration factors R_m_, R_b_, R_f,180_, R_if_, R_rf_ J_0_, J_180_, J_180_/J_0_, and V_T_ are summarized in Table 4. Figure 9 compares the values of R_m_, R_b_, R_f,180_, R_if_, and R_rf_ in a bar graph. Table 4 and Figure 9 show that the R_f,180_ value was a maximum value of 0.393 × 10^9^ kg/m^2^s in the MF process (MF), and it decreased as the process was more complex—from the MF to the MF+PES+UV process. In addition, J_180_ and V_T_ had minimum values of 1072 L/m^2^h and 11.6 L, respectively, and J_180_ increased as the process became more complex. However, V_T_ maximized in the MF+UV process and decreased slightly as the process became more complex. This result is due to the fact that as the process was more complex—from the MF to MF+PES+UV process—the organic matter was effectively removed due to the effects of adsorption and photooxidation, and membrane fouling was controlled. The reason for the different trends in total filtration volume V_T_ seems to be that the initial permeate flux J_0_ is the maximum value in the MF+UV process.

**Figure 10:**
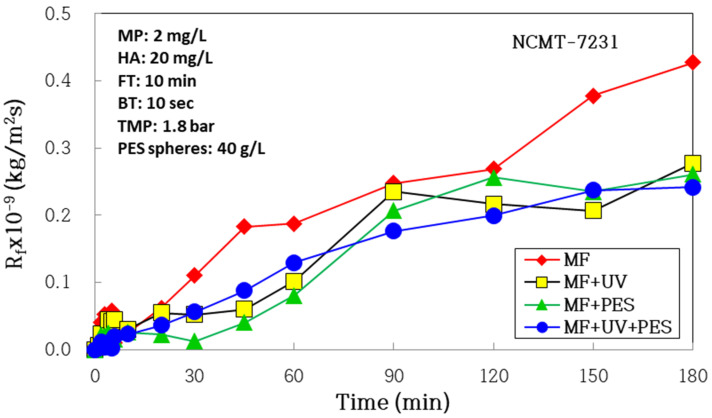
Roles of MF, adsorption, and photooxidation on resistance of membrane fouling in the hybrid process of tubular ceramic MF and TiO_2_-mounted PES spheres at PE (MPs) 2 mg/L and HA 20 mg/L.

Figure 10 shows a graph showing the change in R_f_ for various processes over operating time. The R_f_ was highest for 180 min of operation time when MF was applied alone, and the R_f_ value was maintained at a low value in the MF+PES+UV process. However, the R_f_ values were lower in the 20–60 min interval of the MF+PES process and in the 45–60 min and 150 min intervals of the MF+UV process than those in the MF+PES+UV process. These results indicate that the more complex the process, the higher the removal effect of organic substances via photocatalytic adsorption and photooxidation and, therefore, the lower the membrane fouling.

**Figure 11:**
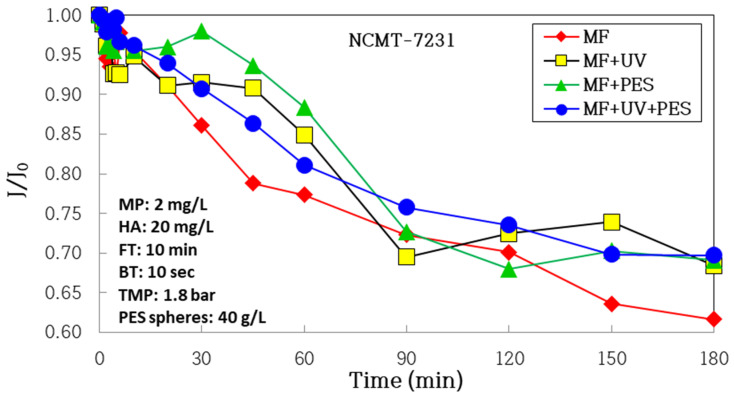
Roles of MF, adsorption, and photooxidation on dimensionless permeate flux in the hybrid process of tubular ceramic MF and TiO_2_-mounted PES spheres at PE (MPs) 2 mg/L and HA 20 mg/L.

Figure 11 shows the change in dimensionless permeate flux J/J_0_ for each process over operating time. At the beginning of operations, there was no significant difference between the different processes and there was no steady trend, but as time passed, the J/J_0_ value remained high in the MF+PES+UV process. Of course, there was a section in the MF+PES process and the MF+UV process that showed a temporarily high J/J_0_ value. However, in general, as the process was simplified from MF+PES+UV to MF, the membrane fouling increased and the permeate flux value decreased, resulting in the lowest J/J_0_ value in the MF-alone process.

When the MP concentration is 2 mg/L and the HA concentration is 20 mg/L, the treatment efficiency of MPs (turbidity) and DOM (UV_254_ absorbance) in the MF, MF+UV, MF+PES, and MF+PES+UV processes are summarized in Table 5 and Table 6, respectively. Table 6 shows that the average turbidity of feed and treated water was 13.0~16.8 NTU and 1.051~1.255 NTU, respectively. As the process was complicated from the MF-alone to the MF+PES+UV process, the treatment efficiency of MPs tended to increase slightly. A protein-based porous N-TiO_2_ semiconductor, which was developed for the removal of MPs, was used to degrade HDPE, one of the MPs [20]. However, under the experimental conditions of this study, the removal effect of MPs via adsorption and photooxidation using a photocatalyst was not clearly seen. From these results, it can be seen that the TiO_2_-mounted PES spheres used in this study were developed to remove DOM, so there are limitations in removing MPs.

Table 6 shows that the average UV_254_ absorbance, which was a measure for the DOM (HA), of the feed water was almost constant, and the average UV_254_ absorbance of the treated water decreased significantly from 0.023 cm*^−^*^1^ to 0.007 cm*^−^*^1^ as the process was complexed. Therefore, the more complex the process in the order of MF+PES+UV, MF+PES, MF+UV, and MF, the greater the treatment efficiency of the DOM increased from 29.1% to 79.7%. From these results, it was confirmed that the DOM was effectively removed via the adsorption and photooxidation of TiO_2_-mounted PES spheres.

Table 7 summarizes the treatment fractions of membrane filtration, adsorption, and photooxidation by TiO_2_-mounted PES spheres obtained by sequentially subtracting the turbidity and DOM treatment efficiency of the MF, MF+UV, MF+PES, and MF+PES+UV processes, which was operated at MP 2 mg/L and HA 20 mg/L. Periodic air backwashing was performed to suppress membrane fouling in all processes. The treatment fraction of MPs identified via turbidity was 90.2%, being the most dominant in the MF process.

However, the treatment fraction of UV-induced photooxidation and adsorption by TiO_2_-mounted PES spheres was weak, at 0.5% and 2.3%, respectively. In addition, in the presence of TiO_2_-mounted PES spheres, there was no fraction of treatment by photooxidation at all. However, the DOM treatment fraction calculated using the treatment efficiency of the UV_254_ absorbance was the highest in the presence of TiO_2_-mounted PES spheres, with a treatment fraction of 46.9%. Next, the treatment fraction of membrane filtration showed a high value of 29.1%. However, the treatment fractions of adsorption by TiO_2_-mounted PES spheres and UV photooxidation without the PES spheres were low at 3.7% and 3.5%, respectively. These results show that the photooxidation effect caused by the UV irradiation of the TiO_2_-mounted PES spheres played a greater role in processing the DOM than membrane separation or adsorption by the PES spheres. Since the TiO_2_-mounted PES spheres used in this study were developed to remove DOMs such as HA, it was found to be ineffective in removing MPs.

#### 3.2.2. Impact of Air Backwashing Cycle (FT, Filtration Time)

We examined the impact of FT in the hybrid water treatment processes of ceramic microfiltration and TiO_2_-mounted PES spheres. After fixing the concentration of MPs at 2 mg/L, the concentration of HA at 20 mg/L, the concentration of PES spheres at 40 g/L, and the backwashing time (BT) at 10 s, the impacts of FT were inspected in terms of R_f_, J/J_0_, and V_T_ by changing the FT to NBF (no backwashing), 6, 8, 10, 20, and 30 min, respectively.

**Figure 12:**
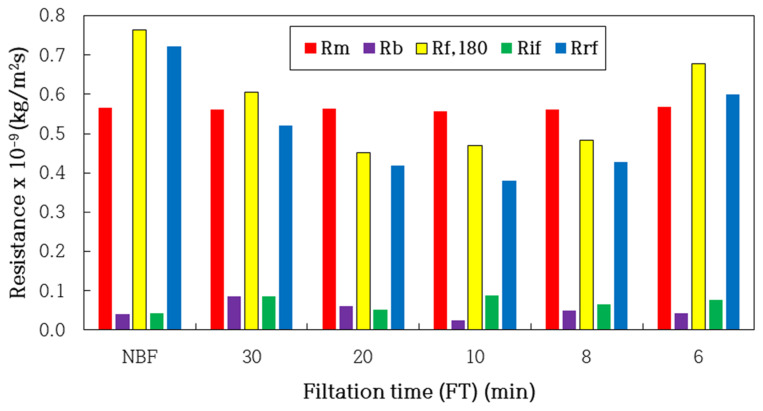
Impact of air filtration time (FT) on filtration resistances for hybrid process of tubular ceramic MF and TiO_2_-mounted PES spheres.

The filtration factors are summarized in Table 8, and Figure 12 compares the values of filtration resistances in a bar graph. By adjusting the initial permeate flux to a certain extent, the resistance of membrane R_m_ showed approximately the same value. The resistance of the boundary layer, R_b_, showed a minimum value at an FT of 10 min and a maximum one at an FT of 30 min. These results prove that an FT of 10 min is the optimal air backwashing interval to suppress the boundary layer in the scope of this study.

As shown in Figure 12, the final resistance of membrane fouling, R_f,180_, was the lowest at 0.451 × 10**^−^**^9^ kg/m^2^s, and the final permeate flux, J_180_, was highest at 650 L/m^2^h at an FT 20 min. The resistance of reversible membrane fouling, R_rf_, showed a minimum of 0.381 × 10**^−^**^9^ kg/m^2^s at an FT of 10 min. As shown in Table 8, the V_T_, which is the total filtration volume, was able to obtain the maximum treated water at 11.51 L at an FT of 10 min. In summary, these results suggest that the optimal FT condition is 10 min to obtain the maximum treated water (V_T_).

**Figure 13:**
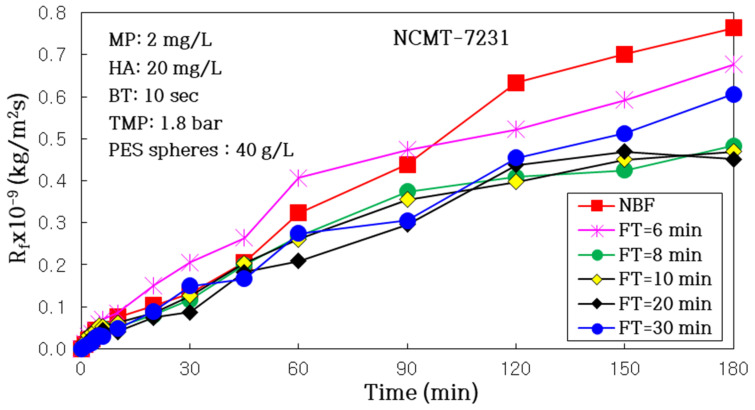
Impact of air backwashing cycle (FT) on resistance of membrane fouling in the hybrid process of tubular ceramic MF and TiO_2_-mounted PES spheres.

Figure 13 shows the change in R_f_ during a 180 min operation time over the FT. As a result, R_f_ maintained generally low at FT of 20 min during 180 min’s operating time, ultimately resulting in the final lowest R_f,180_ value after 180 min. The R_f_ of an FT of 6 min remained highest until the first 90 min of operation, after which the R_f_ of NBF reversed and became the highest. These results demonstrate that membrane fouling is most severe in NBF conditions without air backwashing and that air backwashing every 20 min is the most effective way to suppress membrane fouling.

**Figure 14:**
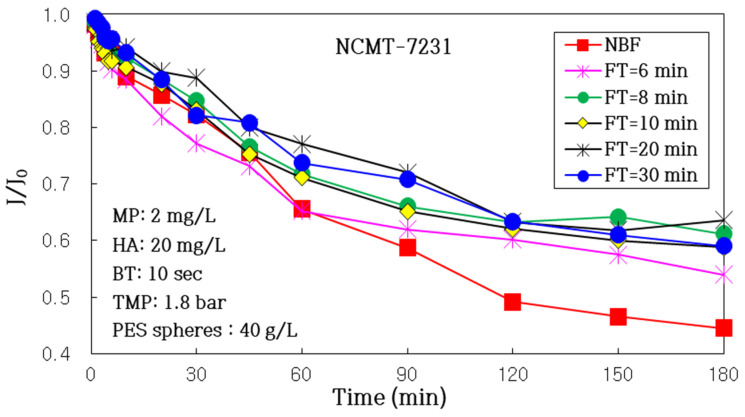
Impact of air backwashing cycle (FT) on dimensionless permeate flux in the hybrid process of tubular ceramic MF and TiO_2_-mounted PES spheres.

Figure 14 shows the evolution of dimensionless permeate flux (J/J_0_) over backwashing cycles during operation. The results showed that under the FT 20 min condition, in which membrane fouling was most effectively suppressed, J/J_0_ generally showed the highest value during the 180 min run and the maximum value at the end of the operations. On the other hand, J/J_0_ at a 6 min FT was minimal until 60 min, after which J/J_0_ in the NBF condition decreased sharply.

**Figure 15:**
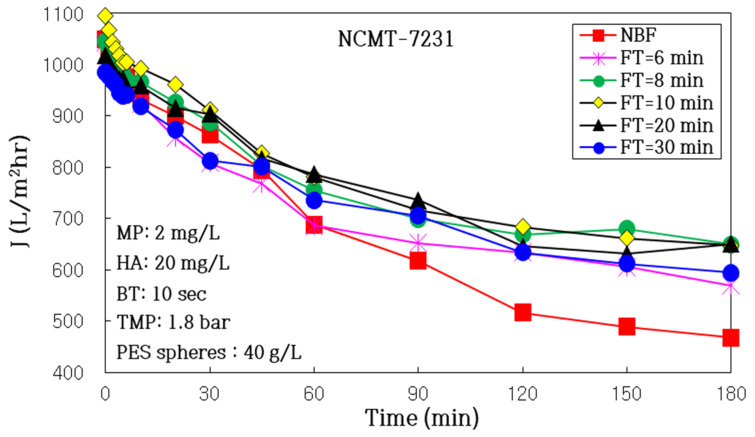
Impact of air backwashing cycle (FT) on permeate flux in the hybrid process of tubular ceramic MF and TiO_2_-mounted PES spheres.

Figure 15 shows the permeate flux (J) value change in the air backwashing cycle (FT) during operation. As Figure 15 shows, 10 min of FT consistently showed the highest value over the course of 180 min of operation, resulting in the highest V_T_ gained. Therefore, 10 min of FT is considered to be the optimal air backwashing cycle condition for the largest filtration volume under the 10 s backwashing time (BT) condition.

**Figure 16:**
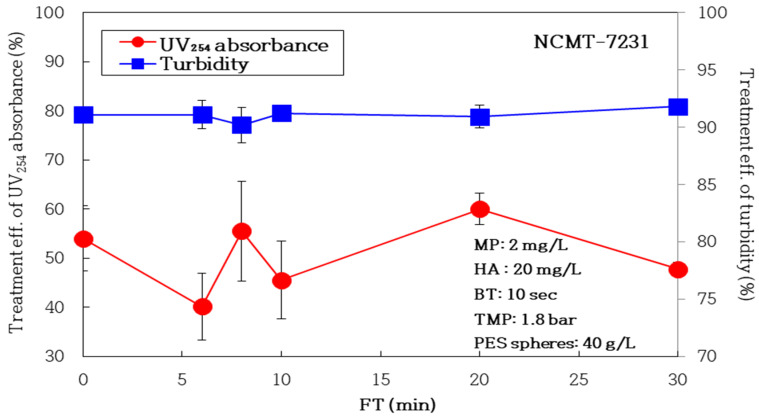
Impact of air backwashing cycle (FT) on treatment efficiency of turbidity and UV_254_ absorbance in the hybrid process of tubular ceramic MF and TiO_2_-mounted PES spheres.

Figure 16 compares the average treatment efficiency of MPs and DOM according to FT through the water quality analysis of turbidity and UV_254_ absorbance, respectively. Looking at Figure 16, the standard deviation is shown by the average treatment efficiency obtained through repeated experiments, and the treatment efficiency at some FT conditions shows unusual trends with different results from experiment to experiment. In conclusion, the average treatment efficiency of MPs was almost constant regardless of FT conditions. However, the DOM treatment efficiency was lowest at 40.2% at 6 min of FT and highest at 60.0% at 20 min of FT.

## 4. Conclusions

In this study, the effects of HA and MP concentrations were examined using a model solution consisting of MPs (polyethylene (PE), 125 μm) and organic matter (humic acid, HA) using only ceramic microfiltration (MF). Next, the role of the adsorption and photooxidation of the TiO_2_-mounted PES spheres was observed in MF, in the MF with the PES spheres without UV irradiation (MF+PES), in the MF of UV irradiation without PES (MF+UV), and in the hybrid process using ceramic microfiltration and PES (MF+PES+UV). Lastly, the membrane filtration characteristics and treatment efficiency were inspected under the influence of the air backwashing cycle (FT) in the MF+PES+UV process. The following conclusions could be obtained from this research.

When examining the effect of organic matter during air backwashing in the MF process, the resistance of membrane fouling (R_f_) was the highest at the maximum concentration of HA 25 mg/L under these experimental conditions. These results show that membrane fouling worsens as the concentration of organic matter increases. Even when the concentration of HA increased, there was no significant difference in the average treatment efficiency (ATE) of the MPs; however, the ATE of DOM tended to decrease. These results imply that high concentrations of DOM limit the removal of it via the MF process alone.

As the concentration of MPs increased, the ATE of the MPs tended to increase slightly; however, the ATE of the DOM showed a clear decreasing trend. As a result, the ATE of the MPs was not significantly related to MP concentration. The reason for the decrease in the ATE of the MPs of the DOM seems to be that as the MP concentration increases, the cleaning effect of the membrane surface occurs, which inhibits the formation of the cake layer.

The R_f,180_ was the maximum value in the MF process, and it decreased as the process was complex from the MF to the MF+PES+UV process. In addition, J_180_ and V_T_ had minimum values and J_180_ increased; however, the total filtration volume (V_T_) maximized in the MF+UV process and decreased slightly as the process became more complex. This result is due to the fact that as the process was more complex from the MF to MF+PES+UV process, organic matter was excellently removed due to the effects of adsorption and photooxidation, and membrane fouling was controlled.

The treatment fraction of MPs was 90.2%, being the most dominant in the MF process. However, the treatment fraction of UV-induced photooxidation and adsorption via the TiO_2_-mounted PES spheres was weak. However, the DOM treatment fraction was the highest in the presence of the PES, with a treatment fraction of 46.9%. Next, the treatment fraction of MF showed a high value of 29.1%. These results show that the photooxidation effect caused by the UV irradiation of the PES played a greater role in removing the DOM than membrane separation or adsorption via PES.

The R_f,180_ was the lowest and the J_180_ was the highest at 20 min of FT. The V_T_ was at its maximum for treated water at an FT of 10 min. These results suggest that the optimal FT condition is 10 min to obtain the maximum V_T_. The ATE of MPs was almost constant regardless of FT. However, the ATE of DOM was highest (60.0%) at 20 min of FT.

## Figures and Tables

**Table 1:** Characteristics of the tubular ceramic MF membrane utilized in this study.

Membrane Model	NCMT-7231
Pore size (μm)	0.1
No. of channels	1
Outer diameter (mm)	8
Inner diameter (mm)	6
Length (mm)	270
Surface area (cm^2^)	50.9
Material	α-alumina
Company	Nano Pore Materials (Seoul, Korea)

**Table 2:** Effect of humic acid concentration on average filtration factors for the process of tubular ceramic MF (NCMT-7231) with air backwashing.

HA (mg/L)	10	15	20	25
R_m_ × 10^−9^ (kg/m^2^s)	0.479	0.667	0.579	0.624
R_b_ × 10^−9^ (kg/m^2^s)	0.021	0.080	0.046	0.217
R_f,180_ × 10^−9^ (kg/m^2^s)	0.139	0.388	0.422	0.465
R_if_ × 10^−9^ (kg/m^2^s)	0.247	0.032	0.108	0.154
R_rf_ × 10^−9^ (kg/m^2^s)	0.123	0.356	0.334	0.311
J_0_ (L/m^2^h)	145	163	183	146
J_180_ (L/m^2^h)	247	213	175	189
J_180_/J_0_	0.589	0.754	0.739	0.772
V_t_ (L)	2.85	2.54	2.24	2.10

**Table 3:** Impact of microplastic concentration on average filtration factors for the process of tubular ceramic MF with air backwashing.

HA (mg/L)	20				
MP (mg/L)	0.5	1	2	5	15
R_m_ × 10^−9^ (kg/m^2^s)	0.619	0.579	0.585	0.632	0.594
R_b_ × 10^−9^ (kg/m^2^s)	0.032	0.046	0.117	0.149	0.098
R_f,180_ × 10^−9^ (kg/m^2^s)	0.341	0.274	0.359	0.492	0.315
R_if_ × 10^−9^ (kg/m^2^s)	0.153	0.163	0.013	0.046	0.032
R_rf_ × 10^−9^ (kg/m^2^s)	0.188	0.111	0.347	0.446	0.283
J_0_ (L/m^2^h)	165	186	168	142	178
J_180_ (L/m^2^h)	244	256	226	203	230
J_180_/J_0_	0.678	0.732	0.743	0.697	0.768
V_t_ (L)	2.24	2.86	2.38	2.19	2.53

**Table 4:** Roles of MF, adsorption, and photooxidation on average filtration factors in the hybrid process of tubular ceramic MF and TiO_2_-mounted PES spheres at PE (MPs) 2 mg/L and HA 20 mg/L.

Process	MF	MF+UV	MF+PES	MF+UV+PES
R_m_ × 10^−9^ (kg/m^2^s)	0.583	0.524	0.530	0.532
R_b_ × 10^−9^ (kg/m^2^s)	0.025	0.010	0.013	0.013
R_f,180_ × 10^−9^ (kg/m^2^s)	0.393	0.277	0.261	0.242
R_if_ × 10^−9^ (kg/m^2^s)	0.035	0.045	0.008	0.015
R_rf_ × 10^−9^ (kg/m^2^s)	0.358	0.232	0.253	0.227
J_180_ (L/m^2^h)	1072	1190	1171	1165
J_0_ (L/m^2^h)	676	817	810	811
J_180_/J_0_	0.624	0.685	0.691	0.697
V_T_ (L)	11.6	13.8	13.7	13.5

**Table 5:** Water quality and treatment efficiency of turbidity (MPs) in the hybrid process of tubular ceramic MF and TiO_2_-mounted PES spheres for roles of microfiltration, adsorption, and photooxidation.

Experimental Condition		Turbidity (NTU)				Average Treatment Efficiency (%)
HA (mg/L)	Process	Feed Water		Treated Water		
		Range	Average	Range	Average	
20	MF+PES+UV	15.0–18.6	16.8	1.130–1.330	1.225	92.5
	MF+PES	13.2–14.7	13.9	0.880–1.350	1.051	92.5
	MF+UV	8.1–16.0	13.5	1.130–1.270	1.180	90.7
	MF	11.3–13.9	13.0	1.120–1.580	1.255	90.2

**Table 6:** Water quality and treatment efficiency of UV_254_ absorbance (HA) in the hybrid process of tubular ceramic MF and TiO_2_-mounted PES spheres for roles of microfiltration, adsorption, and photooxidation.

Experimental Condition		UV_254_ Absorbance (cm^−1^)				Average Treatment Efficiency (%)
HA (mg/L)	Process	Feed Water		Treated Water		
		Range	Average	Range	Average	
20	MF+PES+UV	0.023–0.045	0.034	0.001–0.010	0.007	79.7
	MF+PES	0.023–0.045	0.033	0.017–0.027	0.023	32.8
	MF+UV	0.020–0.037	0.025	0.013–0.018	0.016	32.6
	MF	0.025–0.049	0.034	0.016–0.027	0.023	29.1

**Table 7:** A fraction of treatment efficiencies of MF, adsorption, and photooxidation in the hybrid process of tubular ceramic MF and TiO_2_-mounted PES spheres.

Fraction of Treatment Efficiency	MPs (Turbidity)	HA (UV_254_ Absorbance)
Membrane filtration (%) (MF)	90.2	29.1
Adsorption (%) (MF+PES)-(MF)	2.3	3.7
UV photooxidation (%) (MF+UV)-(MF)	0.5	3.5
Photooxidation with PES beads (%) (MF+PES+UV)-(MF+PES)	0	46.9
Total treatment efficiency (%) (MF+PES+UV)	92.5	79.7

**Table 8:** Impact of air filtration time (FT) on average filtration factors for hybrid process of tubular ceramic MF and TiO_2_-mounted PES spheres (BT 10 s).

FT (min)	NBF	30	20	10	8	6
R_m_ × 10^−9^ (kg/m^2^s)	0.565	0.562	0.563	0.556	0.562	0.568
R_b_ × 10^−9^ (kg/m^2^s)	0.041	0.086	0.062	0.025	0.048	0.042
R_f,180_ × 10^−9^ (kg/m^2^s)	0.764	0.606	0.451	0.470	0.483	0.677
R_if_ × 10^−9^ (kg/m^2^s)	0.043	0.086	0.052	0.089	0.065	0.077
R_rf_ × 10^−9^ (kg/m^2^s)	0.721	0.520	0.419	0.381	0.427	0.600
J_0_ (L/m^2^h)	1049	985	1018	1095	1046	1043
J_180_ (L/m^2^h)	467	594	650	647	649	570
J_180_/J_0_	0.445	0.590	0.636	0.588	0.612	0.540
V_t_ (L)	9.96	10.88	11.37	11.51	11.23	10.32

## Data Availability

Data is unavailable due to privacy or ethical restrictions.
